# A Rare Cause of Childhood Cerebellitis-Influenza Infection: A Case Report and Systematic Review of Literature

**DOI:** 10.1155/2017/4039358

**Published:** 2017-02-20

**Authors:** Şule Gökçe, Zafer Kurugol, Aslı Aslan

**Affiliations:** ^1^Ege University Medical Faculty, Department of Pediatrics, General Pediatrics Unit, Ege University, Bornova, Izmir, Turkey; ^2^Ege University Medical Faculty, Department of Pediatrics, Division of Pediatric Infection Disease, Ege University, Bornova, Izmir, Turkey; ^3^Ege University Medical Faculty, Department of Pediatrics, Ege University, Bornova, Izmir, Turkey

## Abstract

Acute cerebellitis is a benign neurologic condition generally caused by viral or bacterial infections. Influenza associated cerebellitis is extremely rare; a 6-year-old boy with acute cerebellitis, who presented with fever, vomiting, weakness, febrile seizure, and acute cerebellar features, is discussed in this article.

## 1. Introduction

Acute cerebellitis [AC] is an inflammatory process in the cerebellum. The clinical manifestations consist of mild or high-grade fever, headache, altered mental state, and acute onset of cerebellar symptoms such as truncal ataxia, nystagmus, tremor, dysarthria, and hypotonia [1]. The etiology of acute cerebellitis is usually viral, namely, varicella zoster, mumps, epstein-Barr virus, chickenpox, enteroviruses, cytomegalovirus, Q fever, measles, rubella, herpes simplex, rotavirus, and echovirus. AC has also been associated with* Salmonella typhi*,* Bordatella pertussis*,* Borrelia burgdorferi*,* Mycoplasma*,* Coxiella burnetii*, group A* Streptococcus*, and* Orientia tsutsugamushi*. Similar findings have been described in some vaccines [2–6]. It has also been reported to be associated with influenza A and B [7–10]. To our knowledge acute cerebellitis associated with influenza has been diagnosed in a few cases [Online Technical Appendix Table, https://wwwnc.cdc.gov/eid/article/20/9/14-0160-techapp]. In this report, we present a case of acute cerebellitis and mild acute benign encephalopathy associated with seasonal influenza A infection and a brief review of the literature.

## 2. Case Presentation

A previously healthy 6-year-old boy was admitted to our emergency department with fever, vomiting, weakness, ataxia, and febrile seizure. There was no history of neurological disorder or past history of influenza vaccine, drug usage, toxin exposure, or immunization. On admission his physical examination parameters were as follows: weight: 22 kg (50–75 percentile); height: 117 cm (50 percentile); temperature: 38.2°C; heart rate: 110/min; blood pressure: 110/65 mmHg. His condition appeared to be severe. He was conscious, but he suffered from weakness, hypotonia, ataxia, intermittent hallucinations, and vomiting. Clinical and neurologic examination revealed notably broad–based ataxic gait, hypotonia, poor coordination, truncal titubation, positive romberg sign, dysmetria, and dysarthria. Cranial nerve examination produced normal results. The tone and power of the muscles were normal. Deep tendon reflexes were intact, with no signs of meningeal irritation and babinski. Blood testing revealed white blood count: 9350/mm^3^ with neutrophil predominance (68%); hemoglobin: 11,4 g/dL, platelet counts: 251.000/mm^3^, C- reactive protein: 0.1 mg/dL, and sedimentation rate: 21 mm/h. Biochemical investigations, including serum liver/kidney function tests, electrolytes were normal. Computed tomography [CT] scan of the brain revealed no pathologic lesions. Cerebrospinal fluid [CSF] was clear and colorless with an opening pressure of 10 cm of H_2_O and its examination showed normal cell counts; also protein and glucose levels were normal. CSF cultures were bacteriologically sterile. Polymerase chain reaction [PCR] assays of CSF for influenza virus, herpes simplex virus 1 and 2, adenovirus, enterovirus, cytomegalovirus, human herpesvirus- 6, epstein-barr virus, and varicella zoster virus were all negative. His cranial magnetic resonance imaging [MRI] did not show any pathologic signals of the cerebellar hemispheres and gray/white matter. Serologic tests of his blood showed negative results for epstein-barr virus, herpes simplex virus, varicella-zoster virus, cytomegalovirus, measles, mumps, rubella, and mycoplasma pneumoniae. Respiratory viruses such as adenovirus, rhinovirus, respiratory syncytial virus, parainfluenza virus, human bocavirus, human metapneumovirus, and coronavirus were not detected in the nasopharyngeal swab specimen by multiplex PCR. However, we identified influenza A H1N1 virus on the third day of the onset of the symptoms, which was when we started treatment with oseltamivir as 4 mg/kg orally twice a day. The patient was diagnosed with influenza-associated cerebellitis based on the clinical findings. Although the patient's fever and vomiting regressed, his intermittent hallucinations and ataxic gait continued. Therefore, 1 g/kg intravenous immunoglobulin was started on day 4 with a six-hour infusion. After the immunglobulin therapy, the patient improved, and his intermittent hallucinations completely disappeared. He was examined for ataxic gait during his stay in hospital and it was notably resolved without sequelae within 13 days of presentation.

## 3. Discussion

We have described a case of a previously healthy child who developed acute cerebellitis associated with influenza A H1N1. Acute cerebellitis [AC], a cerebellar disorder first described by Westphal and Batten in 1872, is an inflammatory syndrome resulting in acute cerebellar dysfunction [11]. In 2007, the International Multiple Sclerosis Study Group described cerebellitis as a type of clinically isolated syndrome, which can occur as a primary infectious, postinfectious, or postvaccinal disorder [12]. The most frequent clinical features of AC are headache, vomiting, lethargy, altered mental status, coma, ataxia, and fever. Acute cerebellitis maintains an indefinite clinical entity that has been associated with viruses and bacteria. It has also been reported to be associated with influenza A and B [7–10, 13, 14]. However, EBV and VZV appear to be the most frequent pathogens associated with AC [11].

Acute cerebellar ataxia (ACA) is described as a clinical syndrome of acute onset of cerebellar dysfunction, with good long-term prognosis. However, it should be kept in mind that acute cerebellar ataxia and acute cerebellitis overlap considerably, there are no distinct boundaries, and occasionally the two entities can be used in a similar meaning [9, 14–16]. ACA must be differentiated from tumor, abscess, polyneuritis, intoxication, metabolic disease, hereditary degenerative disorders, meningitis, encephalitis, and acute disseminated encephalomyelitis.

Regarding the systematic review of current literature for influenza associated cerebellitis, we searched for articles published up to 2016 in the following databases: Pubmed, Medline, Embase, Cochrane libraries, and CINAHL. We used search terms including “cerebellitis,” “childhood,” “influenza” to identify reports that presented data on influenza-associated cerebellitis. In Pubmed, when we researched the topics about “cerebellitis and childhood”, 27 articles appeared. After excluding the articles which concerned studies of other viruses and radiological studies of AC, we found 10 articles which met our fundamental subject: cerebellitis and influenza infection.

Generally influenza is an acute, self-limited and uncomplicated disease which is caused by influenza A and B and occurs every winter season [17]. The clinical manifestations of uncomplicated influenza infections consist of abrupt onset of fever, headache, myalgia, cough, sore throat, and rhinitis [17, 18]. Central nervous system involvement in influenza is rarely seen and includes a variety of syndromes, more often described in children than in adults. The major clinical entities are encephalitis or encephalopathy [19, 20]. Also, in etiological studies of encephalitis, influenza A and/or influenza B have been identified in up to 10% of pediatric patients [21] while another study by Khandaker et al. [22] showed that 9.7% of all children admitted with influenza had neurological complications, with seizures being the most common neurologic manifestation from the influenza A H1NI virus, followed by encephalitis/encephalopathy. The other neurologic manifestations of influenza are quite varied such as febrile seizures, myositis, cerebellitis, meningitis, meningoencephalitis, and Guillain-Barre Syndrom/Fisher syndrome [18]. At the same time, influenza A H1N1-associated delirium; visual, emotional, and auditory hallucinations; retinal and lateral geniculate nucleus infarctions have also been reported [23, 24].

Influenza associated with cerebellitis is quite rare. A study conducted in the United States showed eight cases of influenza-associated cerebellitis, six of which were children who presented with ataxia, headache, vomiting, dysarthria, and significant bilateral dysdiadochokinesis (Online Technical Appendix Table, https://wwwnc.cdc.gov/EID/article/20/9/14-0160-Techapp1.pdf). Summary of reported cases of influenza-associated cerebellitis in literature was shown in Table 1.

Although viral RNA of influenza viruses is rarely determined in the cerebrospinal fluid [CSF], studies have reported that influenza-associated cerebellitis may occur with adaptive immune responses during the influenza infection due to the fact that increased proinflammatory cytokines have been found in the serum or CSF of patients [25, 26]. Conversely, the RNA of the influenza have been found in cerebrospinal fluid in some previously reported cases of acute cerebellitis. Hayase and Tobita [1] reported a case of a 31-year-old female, with high serum hemagglutination inhibition titer to influenza B and positive CSF polymerase chain reaction [PCR] for influenza B nucleoprotein gene in cerebrospinal fluid, despite normal MR imaging. Sfeir and Najem [10] described an adult case, presenting with ataxic dysarthria and impaired coordination, and influenza RNA was detected in the CSF. In another case, a 6-year-old girl was admitted to hospital with ataxia, and influenza A and influenza B were identifed in CSF with PCR [8]. However, there are some reports that present query AC cases with cerebellar signs, where viral RNA cannot be found in the cerebrospinal fluid [27–29]. For example, a case, reported by Ishikawa et al. [9], presented with fever, headache, and truncal ataxia; and no viral nucleic fragment was detected in CSF. De Bruecker's case of a 4-year-old girl who was admitted to the hospital with a 3-week history of headache, fever, and neck stiffness. Her bronchus aspirate was positive for influenza virus; and viral RNA of influenza was not detected in CSF [30]. Similarly, our case presented with cerebellar signs, and viral RNA could not be isolated from the cerebrospinal fluid. As the nasopharyngeal swab specimen for influenza A H1N1 was positive, this led us to consider influenza-associated cerebellitis.

Diagnosis of AC can be done with history and detailed general/neurological examination. There are no specific markers of diagnosis in blood investigations. AC may be thought of on clinical suspicion after evaluating the differential diagnosis for other serious illnesses such as toxic exposure, infections, and structural problems. In the magnetic resonance imaging [MRI] of AC cases, there are various patterns of cerebellar involvement, and bilateral diffuse hemispheric abnormalities are the most common [30]. There may also be diffuse cortical swelling. Interestingly in some cases MRI is normal. However, none of the MRI findings are pathognomonic for AC. On the other hand, if a patient presents with asymmetric focal deficits and/or altered consciousness, applying MRI should be a necessity [31].

Examination of CSF is not necessary in the diagnosis of AC. As seen in our case, lumbar puncture should be done with findings of central nervous system infection alongside ataxia. Our patient was evaluated with MRI and lumbar puncture because he presented with mild encephalopathy, hallucinations, and seizures together with ataxia. Even though PCR assays of CSF for influenza virus were negative and MRI results were normal, the fact that nasopharyngeal swab specimen was positive and the child had fever together with findings of cerebellitis and encephalopathy led us to think of active influenza infection.

There is no accepted consensus for the treatment of cerebellitis and treatment options. In clinical practice, however, steroid, intravenous immunoglobulin, and antiviral agents have been used in the treatment of patients with acute cerebellitis [32]. Due to the fact that ataxia can be associated with viral encephalitis and bacterial meningitis, antimicrobial therapy should be considered [33, 34]. There is also no consensus on the use of steroids [35]. Kornreich et al. [36] showed in their study that their cases with acute cerebellitis had been empirically treated with steroids, seven had been given antibiotics, and four patients had also been treated with intravenous immunoglobulin. In a case report by Yiş et al. [37], it was stated that an 8-year-old girl who was admitted to their department with vertigo, headache, and vomiting was treated with dexamethasone. In the mentioned case report, the authors suggested that, instead of high pulse methylprednisolone treatment, standard dexamethasone treatment could be a good therapeutic option for mild cases. In an acute life threatening situation such as hydrocephalus in AC, neurosurgical intervention options like external ventricular drainage (EVD), ventricular peritoneal (VP) shunt, and posterior fossa decompression should be performed urgently [38, 39]. As seen in Table 1, many cases with influenza-associated cerebellitis have received antimicrobial therapy and steroid treatment. Tlili-Graiess et al. [4] reported that four cases who were admitted to hospital with headache, fever, vomiting, and ataxia were all treated with prednisone. Our patient was treated with oseltamivir as soon as influenza was detected. Despite the antiviral therapy, however, his neurologic symptoms such as hallucinations and ataxic gait continued. Therefore, intravenous immunoglobulin therapy was given on day 4 and his ataxic gait dissappeared in 13 days.

Acute cerebellitis has good prognosis in childhood. Most patients generally show complete clinical improvement. Among the long-term effects of AC, poor spatial visualization, decreased language skills, and concentration impairment can be seen. Several persistent cerebellar symptoms such as dysmetria, involuntary tremor, and ataxia have also been observed in 10% to 50% of patients [36]. Neither permanent sequelae nor death was reported for these six patients who had influenza-associated cerebellitis as was the case with our patient, except for one patient who developed persistent mild right upper limb paresis, which can be observed on the Online Technical Appendix Table [https://wwwnc.cdc.gov/EID/article/20/9/14-0160-Techapp1.pdf].

## 4. Conclusions

Acute cerebellitis which is an inflammatory process of the cerebellum is a rare, clinically isolated syndrome with varied clinical and radiological features. This neurological disorder has uncertain etiology and heterogeneous pathogenesis. However, AC is mostly considered in association with viral and bacterial infections. To our knowledge, there are few previous reports of acute cerebellitis associated with influenza, but it must be considered in patients who show acute cerebellar features during the influenza season.

## Figures and Tables

**Table 1:** Summary table of reported cases of influenza-associated cerebellitis in literature.

Year	Age/gender	Symptoms	Brain imaging	CSF analysis	Treatment	Outcome
1997 (Hayase and Tobita) [1]	31/F	Fever, ataxia	Normal	Normal	NA	NA

2004 (De Bruecker et al.) [30]	4/F	Headache, fever, and neck stiffness	MRI showed abnormalities in both cerebellar hemispheres and the vermis	Elevated protein concentration and leucocyte cell	NA	NA

2006 (Tlili-Graiess et al.) [4]	4 patients 2-7/F-M	Headache, fever, vomiting, ataxia	In two cases, initial magnetic resonance imaging (MRI) (2 cases) demonstrated increased intensity on T2W and flair sequences of the cerebellar gray matter	High lymphocytes and proteins in samples from 3 children; normal values for 1 child	Prednisone (all patients)	Complete resolution of symptoms in 3 cases; persistent mild right upper limb paresis in 1

2006 (Ishikawa et al.) [9]	25/F	Fever, headache	T2-weighted brain MRI demonstrated a high signal lesion in the cerebellar cortex	Pleocytosis	Oseltamivir	Truncal ataxia normalized after 3 months

2010 (Apok et al.) [7]	14/F	Ataxia	Hydrocephalus	NA	NA	Residual left-sided ataxia after 3 months

2013 (Hackett et al.) [8]	6/F	Headache, worsening dysarthria and ataxia	MRI of the brain confirmed findings consistent with cerebellitis	Normal	Oseltamavir	All symptoms fully resolved after 1 week

2013 (Sfeir and Najem) [10]	37/F	Fever, headache	Brain magnetic resonance imaging (MRI) revealed enlarged bilateral cerebellar hemispheres with evidence of hypointensity	Pleocytosis	Oseltamivir	All symptoms fully resolved after 2 months
