# Corrigendum to “A Rare Cause of Childhood Cerebellitis-Influenza Infection: A Case Report and Systematic Review of Literature”

**DOI:** 10.1155/2018/5781843

**Published:** 2018-02-20

**Authors:** Şule Gökçe, Zafer Kurugol, Aslı Aslan, Candan Çiçek

**Affiliations:** ^1^Ege University Medical Faculty, Department of Pediatrics, General Pediatrics Unit, Ege University, Bornova, Izmir, Turkey; ^2^Ege University Medical Faculty, Department of Pediatrics, Division of Pediatric Infection Disease, Ege University, Bornova, Izmir, Turkey; ^3^Ege University Medical Faculty, Department of Pediatrics, Ege University, Bornova, Izmir, Turkey; ^4^Virology Laboratory, Ege University Medical Faculty, Department of Medical Microbiology, Ege University, Bornova, Izmir, Turkey

In the article titled “A Rare Cause of Childhood Cerebellitis-Influenza Infection: A Case Report and Systematic Review of Literature” [1], Dr. Candan Çiçek was missing from the authors' list. The corrected authors' list is shown above.

Additionally, there were errors in the Case Representation section which should be corrected as follows:

- “CSF cultures were bacteriologically sterile. Polymerase chain reaction [PCR] assays of CSF for influenza virus, herpes simplex virus 1 and 2, adenovirus, enterovirus, cytomegalovirus, human herpesvirus- 6, epstein-barr virus, and varicella zoster virus were all negative” should be corrected to “Multiplex polymerase chain reaction [PCR] assays of CSF for herpes simplex virus 1 and 2, adenovirus, enterovirus, cytomegalovirus, human herpesvirus-6 and -7, Epstein-Barr virus, varicella zoster virus, parechovirus, parvovirus B19 (Neuro 9 Detection, Fast Track Diagnostic, Malta) and influenza virus type A and B, parainfluenza virus, adenovirus, respiratory syncytial virus, human metapneumovirus, human bocavirus, human coronavirus, enterovirus, and rhinovirus (Allplex Respiratory Panel Assays, Seegene, South Korea) were all negative.”
- “Serologic tests of his blood showed negative results for epstein-barr virus, herpes simplex virus, varicella-zoster virus, cytomegalovirus, measles, mumps, rubella, and mycoplasma pneumoniae. Respiratory viruses such as adenovirus, rhinovirus, respiratory syncytial virus, parainfluenza virus, human bocavirus, human metapneumovirus, and coronavirus were not detected in the nasopharyngeal swab specimen by multiplex PCR. However, we identified influenza A H1N1 virus on the third day of the onset of the symptoms, which was when we started treatment with oseltamivir as 4  mg/kg orally twice a day. The patient was diagnosed with influenza-associated cerebellitis based on the clinical findings” should be corrected to “Serologic tests of his blood showed negative results for Epstein-Barr virus, herpes simplex virus, varicella zoster virus, cytomegalovirus (Vidas®, bioMerieux, France), measles, mumps, rubella, and mycoplasma pneumoniae (Diesse Chorus ELISA, Italy). Respiratory viruses including adenovirus, rhinovirus, respiratory syncytial virus, parainfluenza virus, human bocavirus, human metapneumovirus, and coronavirus were not detected in the nasopharyngeal swab specimen by multiplex PCR (Allplex Respiratory Panel Assays, Seegene, South Korea).”
